# Deconstruction of farm machine-related safety interventions: a systematic review and narrative synthesis

**DOI:** 10.1093/annweh/wxae105

**Published:** 2025-01-13

**Authors:** Aswathi Surendran, Jennifer McSharry, Rossella Di Domenico, David Meredith, Oonagh Meade, Sandra Malone, Denis O’Hora

**Affiliations:** School of Psychology, University of Galway, University Road, Galway H91 TK33, Ireland; School of Psychology, University of Galway, University Road, Galway H91 TK33, Ireland; School of Psychology, University of Galway, University Road, Galway H91 TK33, Ireland; Teagasc, Oak Park, Carlo R93 XE12, Ireland; School of Psychology, University of Galway, University Road, Galway H91 TK33, Ireland; Independent Researcher, Australia; School of Psychology, University of Galway, University Road, Galway H91 TK33, Ireland

**Keywords:** BCT, behaviour change interventions, farm injuries, farm safety, occupational safety, tractor rollover, tractor safety

## Abstract

**Introduction:**

Agricultural workplaces have a high number of incidents and fatalities, with the majority occurring from machinery use. Farmers’ behaviour plays a critical role in maintaining safety, as improper or unsafe practices often lead to injuries and fatalities. This review categorises interventions targeting farm machine safety, examining both the behaviour change techniques (BCTs) used and their reported outcomes to understand how the techniques influence safety practices and outcomes on farms.

**Methods:**

The systematic review is reported in accordance with the Preferred reporting items for systematic reviews and meta-analysis guidelines. Seven electronic databases were searched for relevant studies published before June 2024, and the quality of included studies was assessed using Cochrane risk of bias assessment tools. Analysis of intervention behavioural components was guided by the behaviour change wheel framework and BCT taxonomy (v1). The findings were synthesised using a narrative review.

**Results:**

Nine studies were included and a total of 21 BCTs were identified. The most frequently coded BCTs were 4.1 (instruction on how to perform the behaviour), 10.8 (incentive [outcome]), and 16.3 (vicarious consequences) (each *n* = 6). Reported outcomes included reductions in injury rates, improved adoption of safety devices, implementation of safety measures, and positive shifts in safety norms and perceptions. However, due to variations in intervention design and reporting, assessing the direct impact of specific BCTs on these outcomes proved challenging.

**Discussion:**

The use of BCT taxonomy provided a common language for describing intervention components and enabled the standardisation of intervention content analysis. While patterns were observed regarding the commonly used BCTs, their implementation and outcomes, the heterogeneity and limited details provided by studies limited our ability to discern their effectiveness. Providing (i) greater transparency in reporting active intervention components and (ii) clearer connections between components and specific outcomes, will enable enhanced comparisons of future studies, and facilitate a greater understanding of how to support safe machine-related behaviours on farms.

**What’s Important About This Paper?**

This is the first systematic review to identify and describe the behavioural components included in interventions aimed at reducing machine-related accidents on farms. While a variety of behaviour change concepts were identified in interventions reviewed, the impact was difficult to evaluate owing to variations in intervention design and reporting. Use of behavioural science frameworks can facilitate standardised reporting, evaluation, and dissemination of farm safety interventions.

## Introduction

Farm machines are a leading cause of fatalities in the agricultural sector worldwide (Pyykkönen and Aherin 2012; Molina-Guzmán and Ríos-Osorio 2020). The introduction of farm machinery has made agriculture one of the most dangerous industries, alongside mining, and construction (Oden 2014; Angioloni and Jack 2022). Reports from the International Labour Organisation estimated that at least 210 000 fatalities and 250 million accidents are reported yearly on farms worldwide (Hulshof et al. 2021). Although the causes of fatal incidents vary, farm machinery and vehicles such as tractors and all-terrain vehicles have been identified as major contributors to the majority of agricultural fatalities (Murphy and O’Connell 2017; Sorensen et al. 2017). In addition to the tragic human cost, the economic cost of agricultural injuries has been estimated at 8.3 billion US dollars annually, including medical costs and reduced productivity (Dimple et al. 2021). Of greater concern is the fact that while fatality rates in other industries have reduced with the introduction of safety innovations and regulations (e.g. Directive 89/391/EEC—OSH in EU), fatality rates in agriculture remain high (Byard 2017; Ramos et al. 2021).

Agricultural safety interventions have traditionally relied on the ‘Three Es’ approach—Education, Engineering, and Enforcement—adapted from industrial safety models (Sorensen et al. 2016; Austin Burrow 2017). However, this model faces significant challenges in the agricultural context. Engineering interventions, such as power takeoff (PTO) shaft covers, aim to reduce risks through structural modifications. Yet, farms often operate under a diverse range of work conditions using a wide variety of equipment, making it difficult to apply standardised engineering solutions across different farming operations. Additionally, small farms, which form the majority of agricultural enterprises worldwide, often operate on tight financial margins. Thus implementing engineering solutions become significant and sometimes prohibitive (Murphy and O’Connell 2017; Fargnoli and Lombardi 2019; Surendran et al. 2024). Enforcement of safety regulations is also difficult in agriculture due to the decentralised nature of farm work. Many farms are sole-operated or small, making regulatory oversight and compliance difficult. Lone working conditions further complicate enforcement, as there is little capacity for monitoring safety practices on a daily basis (Cecchini et al. 2013; McNamara et al. 2017; Andarge and Lichtenberg 2020).

Educational interventions, based on the assumption that knowledge improves safety practices, face their own obstacles. Behaviour change is inherently difficult, and in farming, the lack of oversight, diverse working conditions, and reliance on personal judgement make it particularly challenging (Narasimhan et al. 2010; Gallagher 2012). Farmers’ safety attitudes often shift only after serious injury occurs (Murphy and O’Connell 2017; Svennefelt 2019). While farm safety interventions aim to educate and motivate change, it remains unclear which educational strategies are most effective, especially when interventions include multiple components (Irwin and Poots 2015).

To effectively improve farm safety, it is crucial not only to assess the overall success of interventions but also to pinpoint the role of individual components (DeRoo and Rautiainen 2000; Coman et al. 2020). Many interventions provide limited insight into how their specific elements impact farmer behaviour, making it difficult to understand what drives real change. This lack of clarity hinders replication and adaptation, reducing the potential for broader application across diverse farming contexts (Petrea 2001; Gallagher 2012; Sorensen et al. 2016; McCallum et al. 2022). By breaking down and analysing these components, interventions can be refined and adapted, ensuring they target specific behaviours more effectively and are more likely to succeed in real-world settings. A systematic approach to understanding these elements is essential for creating interventions that are not only effective but also scalable and adaptable (Skivington et al. 2021).

**Fig. 1.**
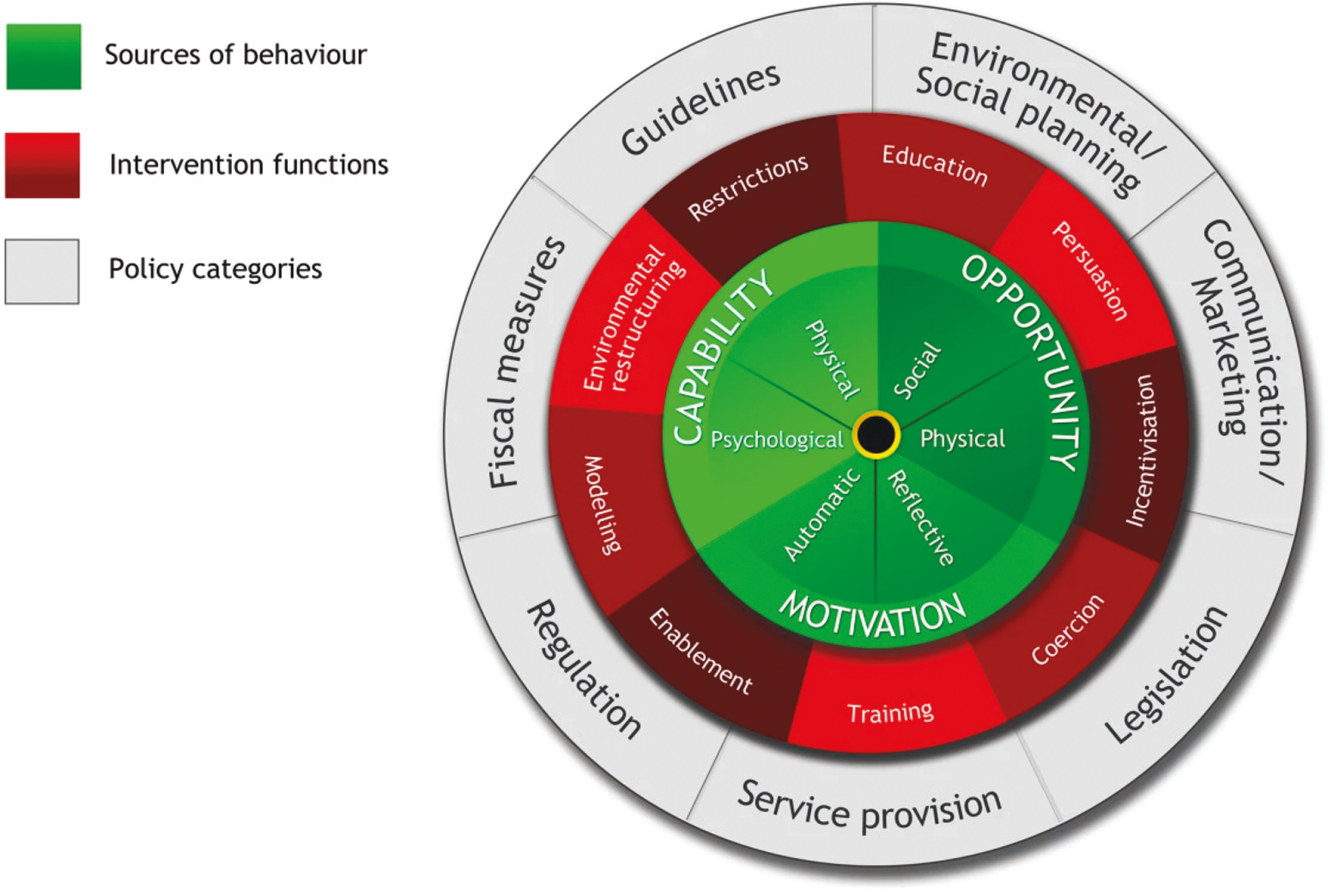
The BCW framework (Michie et al. 2011).

To further explore behaviour change in farm safety, comprehensive frameworks like the behaviour change wheel (BCW) are essential (Michie et al. 2011). The BCW provides a structured approach to understanding behaviour change by identifying key behavioural determinants and mapping them to relevant intervention functions, such as education, persuasion, training, or environmental restructuring (Fig. 1). These functions guide the selection of appropriate behaviour change techniques (BCTs) aimed at driving the desired behaviour change (Michie et al. 2014). BCTs, defined in the BCT Taxonomy (v1), are the observable and replicable components of an intervention that provide a standardised way to describe and analyse its content (Michie et al. 2013).

While BCTs have been used in various fields (French et al. 2014), their application to farm machine safety remains inadequately evaluated. Previous reviews have focussed primarily on outcomes without examining the BCTs embedded in interventions or evaluating how these components influence behaviour. Given the critical need for effective safety practices in agriculture, particularly with high machinery-related fatalities, this review aims to address that gap.

In the present study, we aim to examine three review questions: (i) What intervention types and intervention components have been employed to improve machine-related farm safety?, (ii) What BCTs are utilised within these interventions, and (iii) What are the reported outcomes of these machine-related farm safety interventions?

## Methods

This systematic review was prepared and reported in accordance with the Preferred reporting items for systematic reviews and meta-analysis (PRISMA) guidelines (Moher et al. 2009) and was pre-registered with PROSPERO, the International Prospective Register of Systematic Reviews (registration # CRD42020173834).

### Eligibility criteria

Articles were eligible for inclusion if they met the following inclusion criteria:

(1) The population examined is full or part-time farmers or agricultural workers of any farm type and any gender or farm family members (including children).
(2) The study explicitly described an intervention in which at least one component was explicitly designed to increase farm vehicle or machine-related safety and specifically analysed the impact of the intervention on machine safety outcomes such as injury rate, adoption of safety devices, or personal protective equipment (PPE), behaviour changes, and risk awareness.
(3) The study used an observational or experimental design including randomised controlled trial (RCT), cluster randomised controlled trial, prospective cohort studies with a concurrent control group, quasi-experiments, pre-post- design, longitudinal, correlational, descriptive, or interrupted time-series studies.
(4) Articles published as full texts in peer-reviewed journals.
(5) The study is published in English.

Articles were excluded if they met any of the following criteria:

(1) Studies that did not address machine safety on farms.
(2) Studies that measured the impact of the intervention on the general population, (e.g. school students), and did not report specific effects on the farming population (e.g. farm children).
(3) Interventions for loggers, fishery workers, and hunters (Perez-Lopez 2021).
(4) Other systematic reviews or literature reviews.
(5) Abstracts, conference papers, theses, books, and other grey literature.

### Search strategy

A two-part search strategy was used to identify studies satisfying the inclusion criteria: (i) searched electronic databases using keywords (Cochrane Central Register of Controlled Trials, Cochrane Injuries Group’s specialised register, PubMed, Ovid EMBASE, Ovid PsycINFO, SCOPUS, Ovid EBSCOHOST, and SafetyLit); (ii) searched reference lists of articles included in the review. Systematic reviews and literature from grey sources were excluded; however, the reference lists from these articles were checked to ensure that no relevant papers were missed. Databases were searched in October 2020, and a search update was completed in May 2024 with no time restrictions on the publication date. The search strategy was developed based on the Cochrane review of farm safety interventions by Rautiainen et al. (2008). The search terms were modified based on the search format of individual databases. The search strategy, including keywords, for PubMed is provided in the Table S1. For the search strategies of the other databases, please refer to the BeSafe OSF repository (Surendran *et al.*, 2024).

**Fig. 2.**
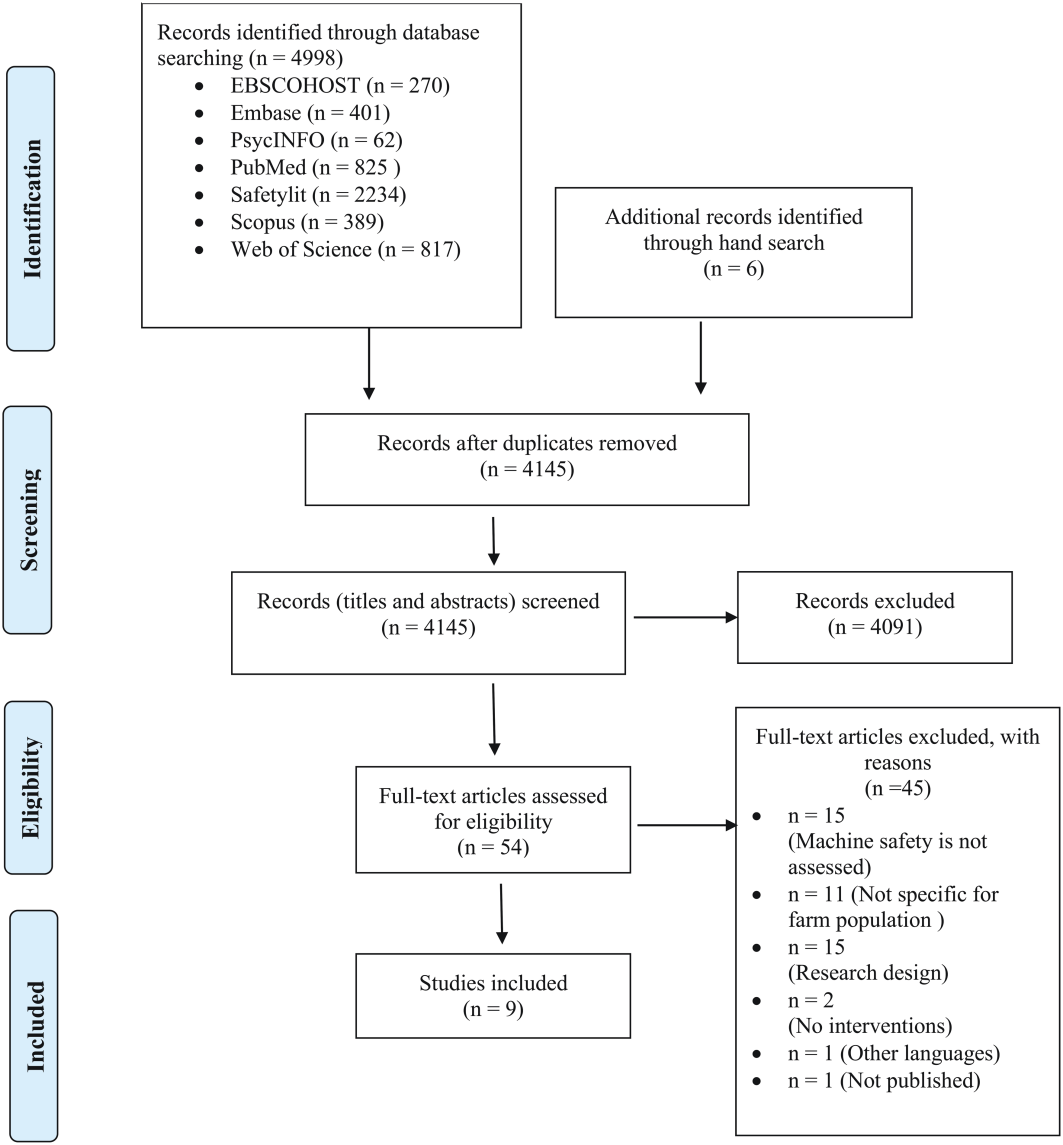
PRISMA flowchart of study selection process.

The PRISMA flowchart in Fig. 2 depicts the searching and screening process. After deduplication, all titles and abstracts were screened against the inclusion and exclusion criteria to identify potentially relevant studies. The second reviewer (RDD) independently reviewed 20% of the titles and abstracts. Articles that met the eligibility criteria at this stage were retrieved for full-text screening, during which the predefined inclusion and exclusion criteria were applied in greater detail. At this stage, the second reviewer independently reviewed 50% of the studies. In June 2024, a search update was conducted by two authors (AS and SM) to ensure the review included the latest studies.

Discrepancies and disagreements were resolved through discussions between the reviewers to reach a consensus on the inclusion of studies. Notably, during this stage, there were discussions regarding including studies targeting school children, as their impact on farm children was initially uncertain. It was decided that these studies would be retained at the screening stage for further examination during the full-text screening phase to verify whether the study explicitly evaluated the impact of the intervention on farm children. Conflicts were resolved with co-authors (DOH, JMS, and OM) where needed. Furthermore, discussions were held to determine whether studies targeting general machine safety adequately evaluated the impact of the intervention on machine safety in the farming context. These discussions aimed to clarify the criteria and ensure that the inclusion and exclusion criteria were consistently applied throughout the screening process. The collaborative approach among the authors helped address uncertainties, clarify the interpretation of criteria, and maintain the rigour of the study selection process.

### Risk of bias assessment

Two Cochrane tools for risk of bias assessment tools were used to assess the bias that affects the reporting of interventions. The Cochrane risk of bias tool for randomised trials (RoB2) assesses the five biases that can affect the reporting of interventions in RCT studies. The Cochrane risk of bias tool for non-randomised trials (ROBINS-NRCI) covers seven domains of bias of non-RCT studies, such as cohort studies, quasi-randomised trials, concurrently controlled studies, and pre-post studies with no control groups. Both tools assign ‘low risk of bias’, ‘some concerns’, or ‘high risk of bias’ for each domain. The overall risk of bias is determined by domain-specific outcomes (Jüni et al. 2016; Higgins et al. 2019). Studies were not excluded from the review if they had poor risk of bias scores. The risk of bias score was used primarily to provide context on the quality of the included studies.

### Data extraction and coding strategy

The data extraction form was developed following the recommendations of the Cochrane Handbook for Systemic Reviews of Interventions (Higgins et al. 2019), and the following data were extracted from the included studies by AS: reference, author, year, population, duration, relevant objectives, intervention details, study design, relevant outcome, and country. Operational definitions of the intervention components, including the intervention categories, sub-categories, and BCW components, are provided in Tables S5–S8.

To answer research question 1 and allow for comparison with previous farm safety reviews (Rautiainen et al. 2008; Nilsson 2016), the interventions were first classified into three primary categories: engineering/technology, education/behaviour change (including incentives), and legislation/enforcement. Financial assistance, such as rebates, was analysed under the educational approach, following the classification provided by the latest Cochrane review on farm safety (Rautiainen et al. 2008). Within each category, interventions were further subdivided into sub-categories based on the specific nature of the intervention.

Following this categorisation, the behavioural components of the interventions were coded using the BCW framework, focussing on intervention functions. The specific BCTs present in the interventions were coded using the BCT Taxonomy v1 (Michie et al. 2013). The first author completed the coding independently using MAXQDA 2022 software (Verbi 2019), and a second reviewer (RDD) independently coded 20% of the studies to ensure reliability. Discrepancies were discussed among the research team (AS, RDD, JMS, and DOH), and a consensus was reached.

As highlighted in the safety literature (Pekkarinen et al. 1994; Stoneman et al. 2014), tools like questionnaires, surveys, and participation incentives, originally designed for data collection or retention, can inadvertently prompt participants to reflect on safety and reconsider their behaviours. Although not designed to modify behaviour, these elements may act as interventions. Therefore, this review coded them as unintended BCTs due to their potential influence.

### Data synthesis

Outcomes were assessed based on various farm safety indicators, as summarised in Table 1. When interventions involved multiple BCW components, the impact of each was analysed independently, as far as the study details allowed. Due to significant heterogeneity in study populations, designs, interventions, and outcome measures, a meta-analysis was deemed unsuitable. Thus, a narrative review, as described by Popay et al. (2006), was used to synthesise the findings.

**Table 1.** Study characteristics.

Reference	Study design	Participants	Intervention(s)	Results^*^
Pekkarinen et al. 1994	Community-randomized controlled trial	Reindeer herders in 53 herding districts in Finland	Intervention Group 1: Information dissemination by theme letters via selected leaders employed by the project Intervention Group 2: Information dissemination during medical examinations conducted by health personnel Control group: No intervention, had access to information about the study the press	Herders reported implementing an average of 5.8 safety measures per herder. The number of helmet users doubled to 5%, and eye/face protector usage increased to 10%. Accident rate decreased from 20 to 15 accidents per 1,000 working days over two years.
Morgan et al. 2002	Pre-Post study	Farmers in the state of Kentucky, USA	Phase-1: Incentives for retrofitting Phase-2: Incentive and ROPS Community based safety capaign	The number of retrofitted tractors with ROPS increased from 4 to 61 after the implementation of the ROPS promotion campaign.
Rasmussen et al. 2003	Randomized controlled trial	Farms in the county of Ringkoebing, Denmark	Intervention Group: Injury registration, safety checks on farms, 1-day farm safety course, and custom safety plans Control group: No intervention	Farmers reported improvement in machinery repairs post-intervention.
Day *et al.*, 2004	Pre-Post study	Full and part-time farmers from the state of Victoria, Australia	Regulatory amendments requiring ROPS installation, a rebate program, and media-based safety campaign	The number of tractors without ROPS decreased from approximately 24% to 7% in the state.
Hallman 2005	Randomized comparative study	350 farms in the state of New York, USA	Offer packages with varying levels of funding for retrofitting ROPS on tractors and free engineering consultation	30 farms accepted the subsidy and retrofitted the ROPS.
Gadomski et al. 2006	Randomized controlled trial	Farm children between 7 and 16 years old employed at New York State farms	Farm visits, telephone injury surveillance, tailored age-related NAGCAT guidelines, and reminders	No difference in the increase in the number of retrofitted tractors. Intervention farms reported less violation in recommended minimum age guidelines on using ATVs and tractors.
Sorensen et al. 2011	Quasi-Randomized controlled trial	Small-scale crop and livestock farms in the state of New York, USA	Intervention Group 1: Rebates and Toll-free hotline assistance Intervention Group 2: Rebates, Toll-free hotline assistance, Social marketing messages, and promotion Intervention Group 3: Toll-free hotline assistance, Social marketing messages and promotion Control group: No intervention	5.1% of the participants retrofitted ROPS The social marketing region reported the greatest increases in readiness to retrofit and intentions to retrofit. Farmers in this region also had higher message recall. Movement from precontemplation to contemplation in farm safety habits was observed in the rebate-only and social marketing regions. In the social marketing region, the mean behavioural intention score increased roughly 4 times the baseline value. Comparisons of changes in subjective norms scores found the most notable increase in the social marketing region, followed by the rebate-only region, the messages and promotion region, and the control region.
Jinnah et al. 2014	Randomized controlled trial	Crop farming families with children aged 10–19 years employed on farms of Georgia state, USA	Intervention Group 1: AgTeen lessons taught by fathers to the children. Intervention Group 2: AgTeen lessons taught by a peer farmer employed by the project to the children. Control group: No intervention.	70% of farmers of parent-led group began using seatbelts on ROPS-equipped tractors, compared to 40% in other groups. 77% of fathers of parent-led group required their youth to wear seatbelts on ROPS-eqipped tractors, compared to 47% in other groups. Fathers of parent-led group showed positive change in perception of injury susceptibility for youth. Youth of parent-led group less likely to operate ROPS tractor without seatbelt compared to control group.
Stoneman et al. 2014	Randomized controlled trial	Crop farming families with children aged 10-19 employed on farms of Georgia state, USA	Intervention Group 1: AgTeen lessons taught by fathers to the children. Intervention Group 2: AgTeen lessons taught by a peer farmer employed by the project to the children. Control group: No intervention.	Fathers from both parent-led and staff-led group were less likely to give youth tractor rides compared to control group. The intervention positively affected the attitudes and injury risk perceptions of both mothers and fathers. Both intervention groups showed a decline in youth giving tractor rides to others post-intervention. After the intervention, parents in the intervention groups demonstrated reduced positive cultural attitudes about extra riding, but many still endorsed its value.

^*^Reported machine safety related outcomes.

## Results

Following the search, 4,998 articles were identified, and 4145 studies were title and abstract screened after deduplication (Fig. 2). A full-text screening was conducted on 54 studies, and nine studies that satisfied the inclusion criteria were included in the review. This included five RCTs (Pekkarinen et al. 1994; Rasmussen et al. 2003; Gadomski et al. 2006; Jinnah et al. 2014; Stoneman et al. 2014), two pre-post intervention studies (Morgan *et al.*, 2002; Day *et al.*, 2004), one randomised comparative study (Hallman 2005) and one quasi-RCT (Sorensen et al. 2011).

### Risk of bias assessment

Tables S2 and S3 summarise the risk of bias assessment for the studies. In the randomised studies, all except one were deemed to have a low risk of bias (Hallman 2005). However, among the non-randomised studies, all studies, except for one quasi-RCT (Sorensen et al. 2011), were deemed to have a high risk of bias. This can be attributed to the fact that the primary objective of these studies was not to evaluate the effectiveness of intervention, but rather to conduct a cost-benefit analysis of a rebate-based intervention (Day *et al.*, 2004) and evaluate the impact of different message types in a campaign-based intervention (Morgan et al. 2002). Therefore, they provided insufficient information relevant to the review.

### Study characteristics

Table 1 provides the general characteristics of the included studies. Table 2 categorises the interventions, identifies their corresponding intervention functions based on the BCW, and lists the specific BCTs identified in each intervention. Additional details are available in Table S5.

**Table 2.** Break down of the intervention categories, sub-categories, and behaviour change components present in the included studies.

Study	Intervention categories	Intervention sub-categories	BCW Intervention Functions identified	BCTs identified
Pekkarinen et al. 1994	Safety education	Safety campaign	Education	2.4 Self-monitoring of behaviour 4.1 Instruction on how to perform the behaviour 9.1 Credible source
Morgan et al. 2002	Safety education	Financial assistance	Incentivisation	10.8 Incentive (outcome)
		Safety Campaign	Education Persuasion Training	4.1 Instruction on how to perform the behaviour 5.1 Information about health consequences 6.1 Demonstration of the behaviour 7.1 Prompts/cues 9.1 Credible source 16.3 Vicarious consequences
Rasmussen et al. 2003	Safety education	Farm audit	Education Persuasion Training Environmental restructuring	1.1 Goal setting (behaviour) 1.2 Problem solving 1.4 Action planning 1.8 Behavioural contract 2.2 Feedback on behaviour 2.4 Self-monitoring of behaviour 2.7 Feedback on outcome(s) of behaviour 4.1 Instruction on how to perform the behaviour 5.1 Information about health consequences 5.2 Salience of consequences 6.1 Demonstration of the behaviour 9.1 Credible source 16.3 Vicarious consequences
Day *et al.*, 2004	Safety education	Financial assistance	Incentivisation	10.8 Incentive (outcome)
		Safety campaign	Education Persuasion Training	4.1 Instruction on how to perform the behaviour 7.1 Prompts/cues 16.3 Vicarious consequences
	Enforcement	Regulation	Restriction	10.11 Future Punishment
Hallman 2005	Safety education	Safety education	Enablement	3.2 Social support (practical)
		Financial assistance	Incentivisation	10.8 Incentive (outcome)
Gadomski et al. 2006	Safety education	Safety education	Education Persuasion Training	1.4 Action planning 4.1 Instruction on how to perform the behaviour 7.1 Prompts/cues
		Farm safety audit	None Identified	2.1 Monitoring of behaviour by others without feedback 2.5 Monitoring outcome(s) of behaviour by others without feedback 2.2 Feedback on behaviour
Sorensen et al. 2011	Safety education	Safety education	Enablement	3.2 Social support (practical)
		Financial assistance	Incentivisation	10.8 Incentive (outcome)
		Promotion	Education Persuasion	5.1 Information about health consequences 5.2 Salience of consequences 7.1 Prompts/cues 9.1 Credible source 16.3 Vicarious consequences
Jinnah et al. 2014	Safety education	Safety demonstration	Education Modelling Persuasion Training	2.1 Monitoring of behaviour by others without feedback 2.4 Self-monitoring of behaviour 4.1 Instruction on how to perform the behaviour 5.1 Information about health consequences 6.1 Demonstration of the behaviour 13.3 Incompatible beliefs 16.3 Vicarious consequences
		Financial assistance	Incentivisation	10.8 Incentive (outcome)
Stoneman et al. 2014	Safety education	Safety demonstration	Education Persuasion Training Modelling	2.1 Monitoring of behaviour by others without feedback 2.4 Self-monitoring of behaviour 5.1 Information about health consequences 5.2 Salience of consequences 6.1 Demonstration of the behaviour 13.1. Identification of self as role model 13.3 Incompatible beliefs 16.3 Vicarious consequences
		Financial assistance	Incentivisation	10.8 Incentive (outcome)

#### Population

The target population for six studies were adult farmers (Pekkarinen et al. 1994; Morgan et al. 2002; Rasmussen et al. 2003; Day et al. 2004; Hallman 2005; Sorensen et al. 2011) while the remaining three studies targeted children from farming families (Gadomski et al. 2006; Jinnah et al. 2014). Five of the nine studies addressed specific farm types, while others were not specific in terms of focus, addressing farming in general. One of those five studies reported that their study included an equal number of participants from all the farm types to eliminate the effect of farm types on the outcome (Rasmussen *et al*. 2003). Of the four remaining studies, Sorensen et al.(2011) targeted livestock and crop farmers, two studies (Jinnah et al. 2014; Stoneman et al. 2014) targeted crop farmers alone, and Pekkarinen et al.(1994) targeted reindeer herders. Interventions were conducted between 1985 and 2014. With the exception of two studies (Morgan et al. 2002; Hallman 2005), all studies provided exact details on the duration of the study, which varied from 1 to 5 y.

#### Theoretical background

Seven studies were explicitly designed to address farm machine-related accidents (Pekkarinen, Anttonen and Pramila 1994; Morgan et al. 2002; Day et al. 2004; Hallman 2005; Sorensen et al. 2011; Jinnah et al. 2014; Stoneman et al. 2014), whereas the remaining two studies addressed machine safety as one of the contributing factors to general farm safety (Rasmussen et al. 2003; Gadomski et al. 2006). Four of the studies were underpinned by one or more theories, including the theory of cognitive dissonance and extended parallel process model (Jinnah et al. 2014; Stoneman et al. 2014), the theory of planned behaviour (Sorensen et al. 2011), and dual coding theory and narrative theory (Morgan et al. 2002). Only two of the studies included input from farmers in the development phase (Morgan et al. 2002; Rasmussen et al. 2003). Four studies were tailored to suit the farm practices (Rasmussen et al. 2003; Gadomski et al. 2006; Jinnah et al. 2014; Stoneman et al. 2014).

#### Outcomes

Across the nine included studies, a diverse range of outcomes was identified, reflecting the heterogeneity in study objectives, designs, population, and measurement strategy (Table 1). While certain outcomes, such as safety perception (*n* = 2), PPE usage (*n* = 2), and roll over protection systems (ROPS) adoption (*n* = 5) were assessed, each study also focussed on specific outcomes aligned with their research objectives. It is important to note that despite examining the same outcomes, variation in measurement approaches was observed across studies. For example, the assessment of retrofit adoption differed across studies regarding the time frame which ranged from monthly to annually. Moreover, data collection methods varied, with some studies using surveys or questionnaires, while others relied on observational data, self-reported data, or rebate reports.

The interventions reported positive behaviour changes related to farm machinery safety (Table 1). Notably, there was increased adoption of safety devices such as, ROPS across five studies (Morgan et al. 2002; Day *et al.*, 2004; Hallman 2005; Sorensen et al. 2011), along with greater usage of PPE such as helmets and eye/face protectors (Pekkarinen et al. 1994; Rasmussen et al. 2003). Improved safety habits and awareness was also observed (Gadomski et al. 2006; Jinnah et al. 2014; Stoneman et al. 2014). Promotion campaigns and incentives for retrofitting ROPS on tractors increased the number of retrofitted tractors (Morgan et al. 2002; Day *et al.*, 2004). Additionally, interventions such as safety courses, injury registration, and custom safety plans contributed to improved machinery repairs and the adoption of safer behaviours on farms (Pekkarinen et al. 1994; Gadomski et al. 2006). Farm safety lessons taught by fathers or peers led to increased seatbelt usage and positive changes in youth’s perception of injury susceptibility (Jinnah et al. 2014; Stoneman et al. 2014). However, despite the immediate reported positive impact on the initial adoption of safety devices, the long-term use and maintenance were unclear and not examined.

#### Intervention categories

Education was the most common intervention approach, with eight of the nine studies using education alone, while the remaining study included both education and enforcement. The educational approach consisted of various strategies (intervention sub-categories) such as financial assistance (*n* = 4) (‘*n*’ refers to the number of studies that employed each specific categories/sub-categories/BCT), safety campaign (*n* = 3), safety demonstration (*n* = 2), farm audit (*n* = 2), and social marketing campaign (*n* = 1). Some studies utilised multiple educational strategies within their interventions, while others focussed on a single approach (Table 2).

### Behavioural content description

**Fig. 3.**
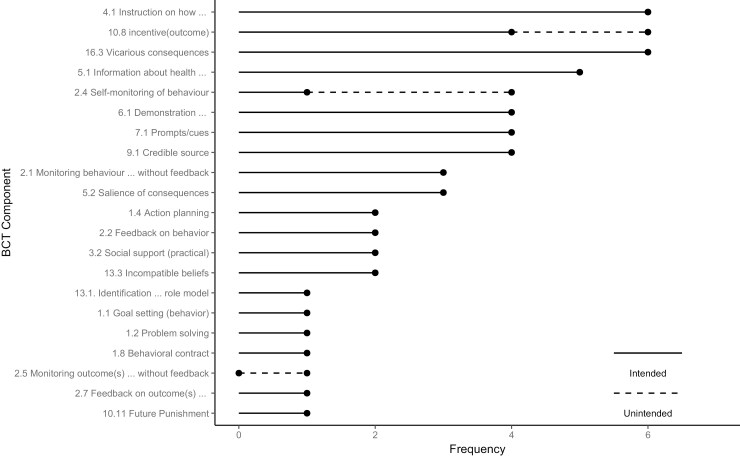
Frequency of the BCTs used in the included intervention studies (*n* = 9).

Overall, 21 unique BCTs were coded in the included interventions, with an average of 6 BCTs (range = 2–13) per study (Fig. 3). The most common techniques were: 4.1 (instruction on how to perform the behaviour), 10.8 (incentive [outcome]), and 16.3 (vicarious consequences) (*n* = 6). Additionally, elements such as participation incentives, surveys, and accident reports, though not originally intended as behaviour change tools, were coded as ‘unintended BCTs’ due to their potential influence on participants’ behaviour by prompting safety reflection.

For example, BCTs 2.4 (self-monitoring of behaviour) and 2.5 (monitoring of outcomes without feedback) were identified in three studies where accident data was collected. Similarly, BCT 10.8 (incentive [outcome]) was present in studies offering participation incentives.

In four studies, all possible BCTs could not be identified due to missing information (Pekkarinen et al. 1994; Morgan et al. 2002; Day *et al.*, 2004; Gadomski et al. 2006). For example, the safety campaign reported in the Victora rebate program (Day *et al.*, 2004) may have included 6.1 (demonstration of the behaviour), 16.3 (vicarious consequences), and 5.1 (information about health consequences), which are included in other campaign programs, however the details of the safety campaign content were not sufficient to confidently assign these BCTs. In addition, certain intervention components were difficult to assign to a specific BCT, even when sufficient information was provided. For example, in this review, rebates were designated as 10.8 (incentives [outcome]). Rebates are typically construed as incentives (Dietz *et al.*, 2009; Bunker *et al.*, 2020; Rand *et al.*, 2020), but it is worth noting that rebates might function, instead, as enabling responses that were prevented by lack of funds (removal of a cost-related barrier to behaviour). If so, then rebates do not motivate behaviour but, rather, provide resources necessary for a behaviour (that is already motivated) to occur.

### Narrative synthesis of interventions

#### Enforcement-based interventions

The enforcement-based intervention (Day *et al.*, 2004) focussed on a regulatory amendment mandating the installation of ROPS on operational tractors. The study reported a reduction in the proportion of unprotected tractors, from 24% to 7%, reflecting improved compliance with safety regulations. The BCT identified was 10.11 (future punishment), as the regulatory amendments had not yet been fully implemented during the intervention. Additionally, a rebate and safety campaign was introduced to promote awareness and provide financial assistance to enhance compliance.

#### Education-based interventions

**Fig. 4.**
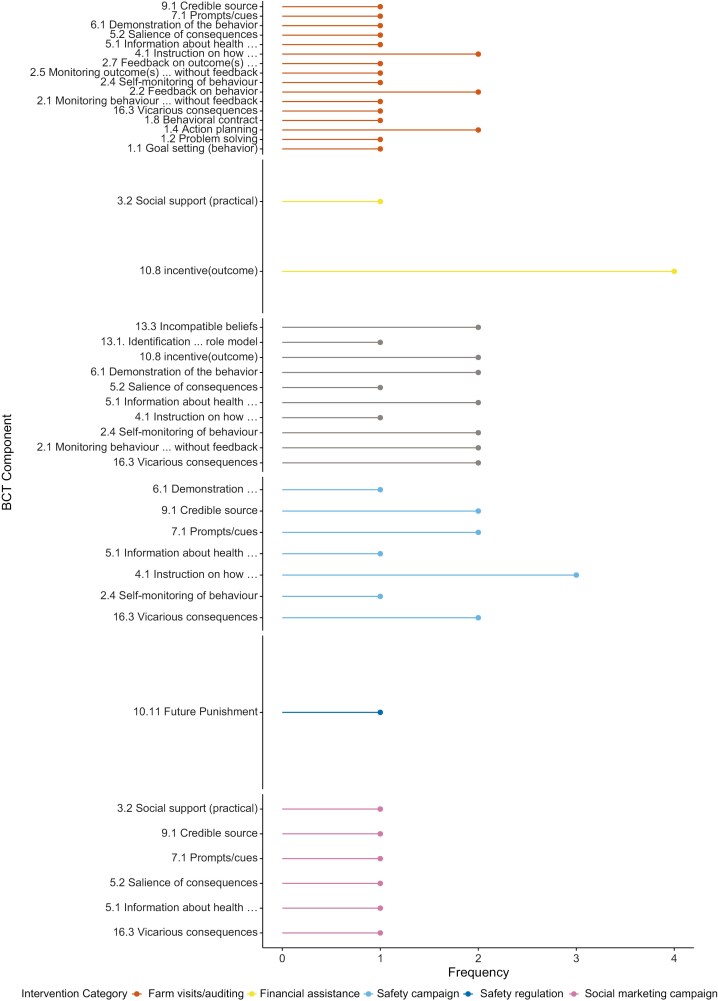
Frequency of each BCT against intervention sub-categories.

Education-based interventions focussed on raising awareness about safety risks, skill development, and the adoption of safety devices. The review identified five sub-categories within these interventions: financial assistance programs, safety campaigns, social marketing campaigns, safety demonstrations, and farm visits/auditing. Similar BCTs were employed across these sub-categories, with financial assistance programmes involving the fewest BCTs, as they primarily provided financial support for ROPS purchase and retrofitting (see Fig. 4). Farm auditing, which included farm visits, training sessions, and feedback, utilised the highest number of BCTs across these interventions.

Four studies incorporated financial assistance as a key component to support ROPS adoption (Morgan et al. 2002; Day *et al.*, 2004; Hallman 2005; Sorensen *et al*. 2011). Three of these studies combined financial incentives with educational components, while Hallman (2005) specifically examined the impact of various rebate rates (0%–100%) on ROPS adoption, identifying 70% as the optimal rebate rate for their local community. Despite clear evidence of the effectiveness of ROPS in preventing fatal rollover injuries and the offer of a full (100%) subsidy, many participants declined to retrofit their tractors, while a small group of farmers retrofitted without any rebate.

In both Hallman (2005) and Sorensen *et al*. (2011), BCT 3.2 (social support [practical]) was operationalised through technical support centres and hotlines. Participants who retrofitted without rebates cited the education and assistance provided through these hotlines as instrumental in their decision-making. The primary focus of these studies was on financial rebates (BCT 10.8 incentives [outcome]).

Moreover, combining financial assistance programs with educational campaigns had a stronger effect on ROPS adoption. Sorensen et al. (2011) reported that while rebates increased participants’ readiness and intention to retrofit, the combination of social marketing and rebates was the most effective. Similarly, Morgan et al. (2002) observed that the introduction of a safety campaign following a subsidy program significantly increased ROPS purchases.

Morgan et al. (2002) further analysed the persuasiveness of narrative-versus statistics-based messages present in the safety campaigns. Although no significant differences in overall effectiveness were observed, statistics-based messages resonated more with participants already inclined to favour safety messages, while narrative-based messages were more persuasive among those initially resistant. The study concluded that messages combining threat, severity, probability, and actionable steps may have a greater long-term impact.

BCT 16.3 (vicarious consequences) was one of the most frequently used technique in education-based interventions, particularly in fear-based approaches. It was often combined with BCTs 5.1 (information about health consequences) and 5.2 (salience of consequences), delivered through testimonials from accident survivors, footage of farm accidents, and videos on the impact of traumatic brain injuries. Instructional techniques such as BCT 4.1 (instruction on how to perform the behaviour) and 6.1 (demonstration of the behaviour) were regularly used to convey actionable safety practices. Regarding the evaluation strategies for campaign-based interventions, Rasmussen et al. (2003) observed that campaigns often focus on general safety information, and it is difficult to find suitable control groups or differentiate the effect of information from other sources such as media or farm websites.

Home-based demonstrations concentrated on specific safety topics, such as teaching safer tractor operations to farm children, using modelling and training. Researchers anticipated that fathers who demonstrated these practices, particularly seatbelt use, might adopt them to reduce cognitive dissonance (BCTs 4.1 [instruction on how to perform the behaviour] and 6.1 [demonstration of the behaviour]). Both studies (Jinnah et al. 2014; Stoneman et al. 2014) reported improvements in farmers’ seatbelt use and risk perceptions. Additionally, youth in the parent-led group were less likely to operate tractors without seatbelts. These findings suggest that peer and family-led safety demonstrations promoted safer tractor behaviours among farmers and youth, potentially supporting intergenerational transmission of safer farm practices.

Three studies examined the impact of the source of safety messages and information. Of these, two (Jinnah et al. 2014; Stoneman et al. 2014), compared the effectiveness of parent-led versus staff-led home-based safety demonstrations targeting children, while the third study (Pekkarinen et al. 1994) evaluated the impact of information disseminated by health professionals, farm personnel, and media among adult reindeer farmers. These studies reported no significant differences in overall effectiveness between the groups. However, they highlighted the challenges, such as information sharing between participants across intervention regions, which could potentially bias the results. In addition, the use of newspapers and digital media to disseminate emotionally charged messages and accident reports assisted in persuading participants to adopt safer practices (Pekkarinen et al. 1994; Rasmussen et al. 2003; Sorensen et al. 2011).

Two studies evaluated the effectiveness of farm safety audits (Rasmussen et al. 2003; Gadomski et al. 2006) and both reported that audits provided prevention strategies tailored to individual farms’ needs. During these visits, safety officers evaluated the farm’s overall health and provided feedback for improving farm machinery handling and safety. Gadomski et al. (2006) highlighted a positive impact on adherence to farm machine-related guidelines for farm children, while Rasmussen et al. (2003) noted improvements in farm machine handling. Notably, these studies did not report increased retrofitting of tractors with ROPS, however, Gadomski et al. (2006) found reduced violations of age guidelines for operating certain farm vehicles. While the audits did employ the common BCTs that were utilised for dissemination in other studies, audits also incorporated BCTs related to customisable and personalised actions such as 1.1 (goal setting [behaviour]), 1.4 (action planning), 1.8 (behavioural contracts), and 2.2 (feedback on behaviour).

In addition to the BCTs previously discussed, 7.1 (prompts/cues) was identified in four studies as a tool to disseminate information or serve as a reminder across various educational sub-categories (Morgan et al. 2002; Day *et al.*, 2004; Gadomski et al. 2006; Sorensen et al. 2011). However, discrepancies arose during the coding of 7.1 (prompts/cues), as intervention descriptions often focussed on delivery methods without detailing the content or how participants engaged with the materials. With more comprehensive descriptions, additional BCTs, such as 5.1 (information about health consequences), 12.1 (restructuring the physical environment), and 12.5 (adding objects to the environment), may have been applicable in some interventions alongside 7.1 (prompts/cues).

Despite not including financial assistance as an intervention component, some studies offered financial incentives to improve participant recruitment and retention (Jinnah et al. 2014; Stoneman et al. 2014). However, none of these studies independently assessed the impact of these incentives on participation and retention rates, leaving their effectiveness undetermined.

## Discussion

This systematic review aimed to address three key questions pertaining to machine-related farm safety interventions. Firstly, it explored the interventions and components utilised to enhance farm safety. Secondly, it identified the BCTs employed within these interventions. Lastly, it examined the outcomes of machine-related farm safety interventions in the context of the BCTs used. There were limits to what can be strongly concluded in terms of the effectiveness of different intervention strategies and BCTs due to (i) the range of intervention components employed concurrently, (ii) the complex interactions among those components affecting safety behaviour, and (iii) difficulties recording safety behaviours and safety failures and (iv) lack of details of the reported interventions. Often, the success of the intervention program varied on a multitude of heterogeneous components concurrently introduced as a part of the program along with confounding variables, and many studies failed to examine the effect of these factors. With these caveats applied to the findings, certain patterns were observed among the reviewed studies. These insights contribute to the broader understanding of enhancing machine safety and provide a foundation for future research and intervention development.

The current review notes that safety education remains a popular approach in machine safety interventions despite engineering and enforcement measures being reported as more effective in safety literature (Fragar and Houlahan 2002). Education-based interventions focussed on raising awareness and skill development, often combining financial assistance programs with campaigns. While the effectiveness of legislation was evident in improving the retrofit of ROPS, installation of safety cabins and use of helmets among farmers, it is essential to combine it with promotional campaigns and financial assistance to increase the knowledge and means to implement it and improve the long-term adaptation (Pekkarinen et al. 1994; Day *et al.*, 2004). The provision of practical social support through hotlines and technical support centres was found to influence participants’ decisions to retrofit ROPS. In conjunction with other educational components, financial assistance programs also increased participants’ readiness and intention to retrofit ROPS. Combining social marketing campaigns with financial incentives yielded the most effective results regarding ROPS purchase rates. The employment of farm audits and home-based farm demonstrations provided opportunities for personalised and customisable actions, and their effectiveness was observed in improving farm safety practices, machine handling, and the physical condition of farms. These findings suggest that no single intervention component alone can comprehensively address the multitude of safety threats in agricultural settings. This echoes the observations from the previous systematic review on farm safety, where multi-faceted interventions encompassing enforcement, engineering, and education were recommended to achieve fundamental changes in farmers’ attitudes and behaviour to stay safe (Rautiainen et al. 2004, 2008; Lee et al. 2017).

This analysis identified various BCTs in the included interventions targeting machine-related farm safety. The most frequent BCTs, such as vicarious consequences and information about health consequences, focussed on changing farmers’ attitudes, knowledge, and behaviours towards safety practices by providing detailed instructions on safety procedures and machine operation as well as the consequences of poor compliance (Fig. 3). Interventions often included detailed explanations of the potential risks and injuries associated with machine-related farm accidents and fatal or near-fatal incidents of farmers. By highlighting the adverse health outcomes and emphasising the importance of safety measures, these interventions aimed to motivate farmers to adopt safer practices. When comparing the efficacy of narratives versus statistics-based messages, it was found that statistics-based messages were more persuasive among participants who already favoured the messages, while narratives were more effective among resistant participants. Another commonly employed BCT was the use of demonstration of the behaviour. This technique aimed to enhance farmers’ understanding and encourage them to replicate the demonstrated behaviours by providing visual examples of safe practices.

Given the popularity of incentives, we recommend further analysis of the optimal rate of financial incentives and their effectiveness in increasing recruitment and retention (Horsburgh and Langley 2011). Although the potential to maintain long-term adherence is demonstrated in various safety intervention programs, we identified a lack of utilising non-monetary-based BCTs, such as 10.4 (social reward), 10.5 (social incentive), 10.7 (self-incentive, 10.9 (self-reward), and 16.2 (imaginary reward) in machine safety literature (Martinsson et al. 2016; Dyreborg et al. 2022).

The systematic review revealed a wide range of outcomes across the included studies, reflecting the diversity in study designs and participant populations. While some interventions focussed on general farm safety (Rasmussen et al. 2003; Gadomski et al. 2006), neglecting high-risk areas such as farm vehicles and machine handling, others specifically targeted machine-related accidents. Positive outcomes were observed in areas such as increased compliance with PPE usage, adoption of safety measures like ROPS and seat belts, and reduced accident rates. However, the lack of standardised outcome measures and inconsistent reporting limited direct comparisons and conclusive assessments of intervention effectiveness. Future research should incorporate standardised measures and evaluate the impact of different intervention components. Furthermore, a comprehensive assessment of outcomes is essential in farm safety interventions. This entails considering behavioural changes, knowledge improvement, safety enhancements, and compliance with regulations. Interventions should adopt a holistic approach to capture the broader impact on the well-being and safety of farmers and the farming community. Despite the outcome variability, these findings highlight the importance of tailored strategies to address specific safety concerns in agriculture and further exploration of the effectiveness of specific intervention components in improving farm safety outcomes.

Demographic factors such as gender, age, location, and farm type play a significant role in farm fatality, however, these factors are often overlooked in research. While it is well known that children and older adults are particularly vulnerable to fatal incidents, there is limited attention given to age-specific concerns in interventions (Nilsson 2016; Murphy and O’Connell 2017). Similar to the previous review (Nilsson 2016), this review found no study that addressed the safety of older farmers. In addition, only one addressed the concern related to the minimum age to handle tractors (Gadomski et al. 2006). Two studies on child safety reported the influence of prevailing norms (Jinnah et al. 2014; Stoneman et al. 2014). Though both studies addressed specific target behaviours concerning children using a similar demonstration approach, due to prevailing cultural attitudes, the demonstration was more effective in increasing the use of safety belts than decreasing the extra riding of youth on tractors. Given the increased likelihood of injuries among children and older farmers, prioritising evidence-based interventions for these target groups is essential. In summary, given that the success of a program relies on addressing relevant barriers and facilitators present in the local context, the review recommends the development of evidence-based interventions that include specific BCTs to address the existing demographic factors, norms, and values.

### Strengths and limitations

The review was limited by a small number of studies focussed on farm machine safety. Including general safety interventions allowed for a more comprehensive sample but led to challenges in making direct comparisons due to diverse interventions and outcome measures. Furthermore, the lack of robust studies led to the inclusion of studies regardless of quality, bias, or sample size, requiring caution in interpreting the findings.

Though more than 50% of the global farm population resides in low-income countries (Forastieri 2000), no studies from these nations were included, possibly due to excluding non-English literature and unpublished articles, leading to potential selection bias.

Previous reviews were often limited to identifying the interventions and measuring their effectiveness, whereas this review attempted to identify the underlying behavioural components, the intervention functions, and BCTs that are commonly employed in farm safety interventions. However, the omission of intervention details in some studies and lack of clarity concerning the intended active ingredients of interventions in others may have resulted in the omission of BCTs. Additionally, due to a lack of understanding of active ingredients and their impact on the measured outcomes, there were limits to what could be strongly concluded regarding BCTs and their effectiveness.

The review supports the findings of previous reviews on the lack of high-quality studies on farm safety interventions, particularly in the challenges encountered from a lack of evaluation in study design and active ingredients. Including supplementary details such as intervention manuals or the TIDieR checklist (Hoffmann et al. 2014) may help communicate the intervention context accurately and improve the transparency, replicability, and generalizability of the study. For future studies, addressing the reporting quality and clearly linking intervention components to specific outcomes will enhance the understanding of the active ingredients driving intervention success and contribute to the development of more tailored and impactful machine safety interventions.

## Conclusions

Previous reviews on farm machine safety interventions have shown that voluntary education programs are the most popular strategy, yet short-term interventions focussed solely on increasing safety knowledge have minimal impact on actual behaviours. This review found that while educational interventions were frequently employed, few studies independently assessed the impact of specific BCTs, underscoring the need for future research to isolate and evaluate the active components within interventions. While some interventions were methodologically sound, many lacked clear theoretical grounding and detailed reporting, which limited replicability and interpretation. To support robust evidence, future interventions should be theoretically driven, target specific demographics, and provide transparent descriptions of all components. Enhanced reporting and rigorous evaluations will allow for meaningful meta-analyses, aiding in the identification of generalisable, scalable solutions for farm machine safety.

## Supplementary material

Supplementary material is available at *Annals of Work Exposures and Health* online.

wxae105_suppl_Supplementary_Tables_S1-S5

## Data Availability

The dataset supporting the conclusions of this article is included within the article. Additional data supporting the project is available in the OSF repository (Surendran *et al.*, 2024).
