# Supplementary material for: Deconstruction of farm machine-related safety interventions: a systematic review and narrative synthesis
Source: Ann Work Expo Health. 2025 Jan 13;69(3):233–50. doi: 10.1093/annweh/wxae105 (PMC11911511; doi:10.1093/annweh/wxae105)
Supplement: wxae105_suppl_Supplementary_Tables_S1-S5 [file wxae105_suppl_supplementary_tables_s1-s5.pdf]

Deconstruction of Farm Machine Related Safety Interventions: A Systematic Review and Narrative Synthesis

Authors: Dr Aswathi Surendran[1], Dr JenniferMcSharry[1], Dr Rossella Di Domenico[1], Dr David Meredith[2], Dr Oonagh Meade[1], Ms Sandra Malone[3], Dr Denis O'Hora[1]

[1]School of Psychology, University of Galway, Ireland

[2]Rural Economy & Development Programme, Teagasc

[3]Independent Researcher, Australia

Supplementary Materials

Table S1: Search strategy used for the PubMed database

Serial No	Search terms*
1	agricultur*[tw] OR farm[tw] OR farms[tw] OR farmer*[tw] OR farming[tw] OR "farm worker"[tw] OR "farm workers"[tw] OR "farmworker"[tw] OR "farmworkers"[tw] OR "farm work"[tw] OR ranch*[tw] OR dairy*[tw] OR dairies[tw] OR greenhous*[tw] OR "green house"[tw] OR orchard*[tw] OR "crop production"[tw] OR harvesting[tw] OR agronom*[tw] OR "Agriculture"[MeSH]
2	tractor* OR quad OR "quad bike" OR "all terrain vehicle" OR atv OR "all-terrain vehicle" OR "farm machinery" OR "machine*" OR mower OR Rake OR Baler OR Augers OR Trailer OR Loader OR excavator OR Teleporter
3	injur*[tw] OR accident*[tw] OR Accidents[MeSH] OR trauma[tw] OR harm*[tw] OR wound*[tw] OR "fall"[tw] OR OR "falling*"[tw] OR "burn"[tw] OR burning[tw] OR "burns"[tw] OR fatal*[tw] OR suffocat*[tw] OR lacerat*[tw] OR asphyxia[tw] OR asphyxiate*[tw] OR "electric shock"[tw] OR Electrocution[tw] OR "power line"[tw] OR Entanglemen*[tw] OR "injuries"[sh] OR "Accidents, Occupational"[Mesh] OR "Wounds and Injuries"[MeSH] OR roll-over[tw] OR "roll over"[tw] OR "equipments"[tw] OR PTO*[tw] OR "power take off"[tw] OR "power take-off"[tw] OR "scald"[tw] OR scalding[tw] OR scalds[tw] OR "crush" OR "collisions" OR amput* OR mutilate* OR fractur* OR "death"
4	safet*[tw] OR prevent*[tw] OR control*[tw] OR risk*[tiab] OR "risk management"[MeSH] OR "accident prevention"[MeSH] OR Safety[MeSH] OR "Safety Management"[MeSH] OR "prevention and control"[sh] OR risk[MeSH] OR intervention[tw] OR "Accident Prevention"[MeSH] OR "on-farm"[tw] OR intervene*[tw] OR "protective gears" OR mitigation[tw] OR education[tw] OR "roll-over protective structures"[tw] OR ROPS[tw] OR "Personal Protective Equipment"[tw] OR "protective gears"[tw] OR "protective guard"[tw] OR "safety check"[tw] OR "PPE"

5	randomized controlled trial[pt] OR controlled clinical trial[pt] OR randomized controlled trials[mh] OR random allocation[mh] OR double-blind method[mh] OR single-blind method[mh] OR clinical trial[pt] OR clinical trials[mh] OR "clinical trial"[tw] OR ((singl*[tw] OR doubl*[tw] OR trebl*[tw] OR tripl*[tw]) AND (mask*[tw] OR blind*[tw])) OR "latin square"[tw] OR placebos[mh] OR placebo*[tw] OR random*[tw] OR research design[mh:noexp] OR comparative study[tw] OR Evaluation Study[tw] OR comparative study[pt] OR Evaluation Study[pt] OR Follow-Up Studies[mh]OR prospective studies[mh] OR cross-over studies[mh] OR Cohort Studies[mh] OR Longitudinal Studies[mh] OR control*[tw] OR prospectiv*[tw] OR volunteer*[tw] NOT (animal[mh] NOT human[mh]
6	effect* [tw] OR control* [tw] OR evaluation* [tw] OR program* [tw]) NOT (animal[mh] NOT human[mh
7	1&2&3&4
8	5&7
9	6&7
10	8 OR 9 limit 16 to (english language

* Search terms for other studies available in the OSF profile (Aswathi et al., 2022).

Table S2: Quality appraisal and Risk of Bias Assessment - Cochrane risk-of-bias tool for randomized trials

Study	Randomisation process	Deviations from intended interventions	Missing outcome data	Measurement of the outcome	Selection of the reported result	Overall
**Gadomski et al., 2006**	+	+	+	+	+	+
**Pekkarinen et al., 1994**	?	+	+	+	+	+
**Jinnah et al., 2014**	+	+	+	+	+	+
**Stoneman et al., 2014**	+	+	+	+	+	+

**Hallman, 2005**	+	+	+	?	+	?
**Rasmussen et al., 2003**	+	+	+	+	+	+

\+ Low risk ? Some concern - High risk

Table S3: Quality appraisal and Risk of Bias Assessment - Cochrane risk-of-bias tool for non-randomised trials

Study	Confounding Bias	Selection Bias	Classification of Interventions	Deviations from intended interventions	Missing outcome data	Measurement of outcomes	Selection of the reported result	Overall
**Sorensen et al., 2011**	?	+	+	+	+	?	?	+
**Morgan et al., 2002**	-	+	+	?	-	-	-	-
**Day et al., 2004**	-	+	+	+	+	+	+	-

+ Low risk ? Some concern - High risk

Table S4: Data extraction table

Authors (year of publication, country)	Name of the study and relevant objective(s)	Study Design	Participants	Intervention(s)	Intervention approaches identified	Quality rating	Theoretical underpinning	Individualised intervention	Participatory research
Pekkarinen et al., 1994<br><br>Finland	Accident Prevention in Reindeer Herding<br><br>To reduce the snow mobile accidents among reindeer farmers	Community-randomised controlled trial<br><br>Time period: 1985-1987	*Targeted*: Reindeer herders in 53 herding districts<br><br>*Sample size*: Intervention A : 18 districts (N=1157)<br>Intervention B : 17 districts (N=1 065)<br>*Control Group*:<br>18 districts	Intervention Group 1 : Information dissemination by theme letters via selected leaders employed by the project<br>Intervention Group 2 : Information dissemination during medical examinations conducted by health personnel<br>Control group: No intervention, had access to information about the study the press<br><br>*Results*: Herders reported implementing an average of 5.8 safety measures per herder.<br>The number of helmet users doubled to 5%, and eye/face protector usage increased to 10%. Accident rate decreased from 20 to 15 accidents per 1000 working days over two years.	Safety education	High	Not Reported	No	No
Morgan et al., 2002<br><br>Kentucky, USA	Stories or Statistics? Farmers' Attitudes Toward	Pre-Post study using surveys	*Targeted*: Farmers in the state of Kentucky, USA	Phase 1: Incentives for retrofitting<br>Phase 2: Incentive and ROPS Community-based safety capaign	Safety education	Low	Dual coding theory<br>Narrative theory	No	Yes

	Messages in an Agricultural Safety Campaign<br><br>To increase the adoption of ROPS and use of seat belts while driving tractor	Time period: Three years	*Sample size*: Farmers from two counties of the state of Kentucky<br><br>*Control group: None*	*Results*: The number of retrofitted tractors with ROPS increased from 4 to 61 after the implementation of the ROPS promotion campaign.					
Rasmussen et al., 2003<br><br>Ringkoebing, Denmark	Prevention of farm injuries in Denmark<br><br>To reduce the work-related accidents and injuries	Randomised controlled trial<br><br>Time period: 1993-1997	*Targeted*: Farms in the county of Ringkoebing, Denmark<br><br>*Sample size*: Intervention group: 99 farmers from approximately equal numbers of dairy, swine, crops and mixed farms.<br><br>*Control group:*102 farmers	*Intervention Group : Injury registration, safety checks on farms, 1-day farm safety course and custom safety plans*<br>*Control group: No intervention*<br><br>*Results*: Farmers reported improvement in the machinery repairs post intervention.	Safety education	High	None	Yes	Yes

Day et al., 2004<br><br>Victoria, Australia	An Australian experience with tractor rollover protective structure rebate programs: process, impact and outcome evaluation<br><br>To increase the adoption of ROPS among farmers with tractor rollover protective structure rebate program	Pre-Post study using surveys<br><br>Time period: 1997-1998	*Targeted*: Full and part-time farmers from the state of Victoria<br><br>*Sample size*: Not available<br><br>*Control Group:* None	A regulatory amendment that required all operational tractors to be fitted with ROPS. A rebate program that offered $AUD 150 to farmers for each tractor retrofitted with ROPS. A safety campaign involving television advertising, mailing of application forms to all Victorian farmers, and information dissemination at farm field days and other public events.<br><br>*Results*: The proportion of unprotected tractors was reduced from approximately 24%–7% in the state.	Regulation Safety education	Low	Not Reported	No	No
Hallman, 2005<br><br>New York, USA	ROPS Retrofitting: Measuring Effectiveness of Incentives and Uncovering Inherent Barriers to Success<br><br>To increase the adoption of ROPS	Randomised comparative study<br><br>Time period: Not available	*Targeted*: Farms in the state of New York, USA<br><br>*Sample size*: Intervention group: 365 Farms<br><br>*Control group:* None	Nine different Offer packages varying from 0% to 100% for retrofitting ROPS on tractors Free engineering consultation.<br><br>*Results*: Out of the 365 farms, 30 farms accepted the subsidy and retrofitted the ROPS. An incentive of 75% to 90% funding attracted the greatest	Safety education	Medium	None	No	No

	among farmers			number of participants per dollar offered.					
Gadomski et al., 2006<br><br>New York, USA	Efficacy of the North American Guidelines for Children's Agricultural Tasks in Reducing Childhood Agricultural Injuries<br><br>Guidelines to reduce the agricultural injuries among children.	Randomised controlled trial<br><br>Time period: 2001-2003	*Targeted*: Farm children between 7-16 years old and employed at farms<br><br>*Sample size*: Intervention group:462 farms<br><br>*Control group:* 469 farms	Intervention Group: Farm visits,<br>Telephone injury surveillance every 3 months,<br>Customised guidelines and remainder mailers and souvenirs<br>Control group: No intervention<br><br>*Results*: No difference between the 2 groups in adding a rollover protection structure and adding or repairing a power takeoff. Intervention farms reported fewer violations in recommended minimum age guidelines on using ATVs and tractors, hitching and un-hitching trailed implements to tractors, and baling hay than control farms.	Safety education	High	Child development principles. No further details available	Yes	No
Sorensen et al., 2011<br><br>New York, USA	The Social Marketing of Safety Behaviours: A Quasi–Randomized Controlled Trial of Tractor	Quasi-Randomised controlled trial<br><br>Time period: 2006-2007	*Targeted*: Small-scale crop and livestock farms in the state of New York, USA<br><br>*Sample size*: Intervention	Intervention Group 1: Rebates and Toll-free hotline assistance<br>Intervention Group 2: Rebates, Toll-free hotline assistance, Social marketing messages and promotion<br>Intervention Group 3: Toll-free hotline	Safety education	High	Theory of planned behaviour	No	Yes

	Retrofitting Incentives<br><br>To increase the adoption of ROPS among farmers		1: 214 participants<br>Intervention 2: 227 participants<br>Intervention 3: 282 participants<br>*Control group*: 383 participants	assistance, Social marketing messages and promotion<br>Control group: No intervention<br><br>*Results:* Eighteen(5.1%) of the final 350 respondents who completed the program reported retrofitting a tractor since the start of the intervention. The social marketing region reported the greatest increases in readiness to retrofit and intentions to retrofit. Farmers in this region also had higher message recall.<br>Movement from precontemplation to contemplation in farm safety habits was observed in the rebate-only and social marketing regions.<br>In the social marketing region, the mean behavioural intention score increased roughly 4 times the baseline value.<br>Comparisons of changes in subjective norms scores found the most notable increase in the social marketing region, followed by the rebate-only region, the messages and promotion region, and the control region.					

Jinnah et al., 2014<br><br>Georgia state, USA	Involving Fathers in Teaching Youth About Farm Tractor Seatbelt Safety: A Randomised Control Study<br><br>AgTeen, a family-based farm safety intervention to increase the use of seat belts while driving tractors	Randomised controlled trial<br><br>Time period: 2007 -2012	*Targeted*: Crop farming families with children aged 10-19 and employed at farm<br><br>*Sample size*: Intervention 1: 47 families , Intervention 2: 53 families *Control group:* 51 families.	Intervention Group 1: AgTeen lessons taught by fathers to the children.<br>Intervention Group 2: AgTeen lessons taught by a peer farmer employed by the project to the children.<br>Control group: No intervention. Data collection forms were provided.<br>Incentives were provided to all the participants.<br><br>*Results*: 70% of farmers of parent-led group began using seatbelts on ROPS-equipped tractors, compared to 40% in other groups.<br>77% of fathers of parent-led group required their youth to wear seatbelts on ROPS-eqipped tractors, compared to 47% in other groups.<br>Fathers of parent-led group showed positive change in perception of injury susceptibility for youth.<br>Youth of parent-led group less likely to operate ROPS tractor without seatbelt compared to control group.	Safety education	High	Theory of Cognitive Dissonance Extended Parallel Processing theory	Yes	No
Stoneman et al., 2014	Changing a Dangerous Rural Cultural Tradition: A	Randomised controlled trial	*Targeted*: Crop farming families with children aged 10-19 and	Intervention Group 1: AgTeen lessons taught by fathers to the children.<br>Intervention Group 2: AgTeen lessons taught by	Safety education	High	Theory of Cognitive Dissonance & Extended Parallel	Yes	No

Georgia state, USA	Randomised Control Study of Youth as Extra Riders on Tractors<br><br>AgTeen, a family-based farm safety intervention to reduce the extra riding on the tractors by children in the farming families	Time period: 2007 -2012	employed at farm<br><br>*Sample size*: Intervention A: 47 families Intervention B : 53 families *Control group:* 51 families.	a peer farmer employed by the project to the children. Control group: No intervention. Data collection forms were provided.<br>Incentives were provided to all the participants.<br><br>*Results*: Fathers from both parent-led and staff-led group were less likely to give youth tractor rides compared to control group. The intervention positively affected the attitudes and injury risk perceptions of both mothers and fathers.<br>Both intervention groups showed a decline in youth giving tractor rides to others post-intervention.<br>After the intervention, parents in the intervention groups demonstrated reduced positive cultural attitudes about extra riding, but many still endorsed its value.			Processing theory cess Model.		

Table S5: Intervention categories and their definition

**Intervention**	**Definition**
Engineering	Preventive measures involving engineering or structural changes to minimise or eliminate the risks.
Education	Preventive measures that involve training, knowledge and skill translation that enable workers to understand safety knowledge and develop safe attitudes.<br>Any form of financial assistance.
Enforcement	Introduction of safety rules and ensuring compliance from farmers through legal enforcement.

Table S6: Intervention sub-categories and their definition

**Intervention**	**Definition**
Farm visits/auditing	A systematic assessment of safety hazards and risks on a farm by farm advisors or safety experts.
Financial assistance programs	Any form of assistance to encourage the adoption of safety practices or equipment.
Safety campaigns	Any form of mass communication campaigns or promotions promote a program or raise awareness
Safety demonstrations	Conducting live demonstrations to showcase safe practices and safety equipment usage on farms.
Social marketing campaigns	Any form of monetary benefits

Table S7: Intervention functions with their definition and contextual details.

**Intervention Function**	**Definition** (Michie et al., 2011)	**Contextual details**
Education	Increasing knowledge or understanding.	Providing information on risks associated with farming, safety guidelines, operation of farm machines etc.
Enablement	Increasing means/reducing barriers to increase capability (beyond education and training) or opportunity (beyond environmental restructuring).	Providing support like hotlines to adopt safety devices like ROPS.
Environmental restructuring	Changing the physical or social context.	Changing the farm layout to reduce the risks. Introducing objects to increase awareness about safety.
Incentivisation	Creating an expectation of incentives for performing desired behaviour.	Provide financial support for participation in the safety programmes, retrofit safety devices etc.
Modelling	Providing an example for people to aspire to or imitate.	Collaborating with family members, peer coaches to educate or demonstrate recommended safety guidelines.
Persuasion	Using communication to induce positive or negative feelings or stimulate action.	Narrating real-life accidents, prompts about own and families' well-being, etc., to increase compliance or participation in the safety initiatives.
Restrictions	Using rules to reduce the opportunity to engage in the target behaviour (or to increase the target behaviour by reducing the opportunity to engage in competing behaviours).	Prohibiting the use of unsafe farm vehicles.
Training	Provide any kind of training to any parties involved in the intervention program.	Providing skill training, risk assessment training etc.

Table S8: Operational Definitions for Assessed Behaviour Change Techniques (BCTs)

**BCT**	**Definition** (Michie et al., 2013)	**Contextual details**
1.1 Goal setting (behaviour)	Set or agree on a goal defined in terms of the behaviour to be achieved.	1. Identifying and agreeing to change selected behaviours.
1.2 Problem solving	Analyse , or prompt the person to analyse, factors influencing the behaviour and generate or select strategies that include overcoming barriers and/or increasing facilitators.	1. Encouraging participants to identify the<br>a) cause of accidents<br>b) Solutions for avoiding accidents
1.4 Action planning	Prompt detailed planning of performance of the behaviour (must include at least one of context, frequency, duration and intensity).	1. Creating guidelines and plans on how to perform a specific task .
1.8 Behavioural contract	Create a written specification of the behaviour to be performed, agreed on by the person, and witnessed by another.	1. Create contracts that specifying that they will complete agreed tasks or goals with peers as witness.
2.1 Monitoring of behaviour by others without feedback	Observe or record behaviour with the person's knowledge as part of a behaviour change strategy.	1. Observing the behaviour by the<br>a) program facilitator<br>b) Safety advisor

2.2 Feedback on behaviour	Monitor and provide informative or evaluative feedback on performance of the behaviour.	1. Feedback provided on the behaviour/practices by<br>a) safety advisors<br>b) program facilitators
2.4 Self-monitoring of behaviour	Establish a method for the person to monitor and record the outcome(s) of their behaviour as part of a behaviour change strategy.	1. Reporting own behaviour via<br>a) Survey<br>b) Questionnaire<br>c) Status report
2.5 Monitoring outcome(s) of behaviour by others without feedback	Observe or record outcomes of behaviour with the person's knowledge as part of a behaviour change strategy.	1. Observing the outcome of the adoption of safety behaviours or risky behaviours by the<br>a) program facilitator<br>b) Safety advisor<br>2. Surveys conducted by the facilitators.
2.7 Feedback on outcome(s) of behaviour	Monitor and provide feedback on the outcome of performance of the behaviour.	1. Observing and providing the feedback on the outcome of the adoption of safety behaviours or the engagement in risky behaviours by<br>a) safety advisors<br>b) program facilitators
3.2 Social support (practical)	Advise on, arrange, or provide practical help for performance of the behaviour	1. Arrange/assist in a) Sourcing machine parts<br>b) repairing the farm machines<br><br>2. Technical consultation

4.1 Instruction on how to perform the behaviour	Advise or agree on how to perform the behaviour	1. Information sessions/knowledge transfer/skill building activities on how to use<br>a) PPE<br>b) Ergonomic ways to perform tasks<br>c) Managing yard etc.
5.1 Information about health consequences	Provide information about health consequences of performing the behaviour	1. Provide information on health consequences<br>a) associated with farm accidents
5.2 Salience of consequences	Use methods specifically designed to emphasise the consequences of performing the behaviour with the aim of making them more memorable	1. Effect of farm accidents and specific consequence on a) physical development<br>b) emotional well being
6.1 Demonstration of the behaviour	Provide an observable sample of the performance of the behaviour, directly in person or indirectly e.g. via film, pictures, for the person to aspire to or imitate	1. Demonstrate a) How to operate the farm vehicles<br>b) Best practices
7.1 Prompts/cues	Introduce or define environmental or social stimulus with the purpose of prompting or cueing the behaviour. The prompt or cue would normally occur at the time or place of performance	1. Send the information to the participant's home via<br>a) Mailers<br>b) Put the application form at the participant's home to fill out<br>2. Add a remainder with safety messages, like calendar/fridge magnets at farm houses.<br>3. Adding posters with information in farm shops.<br>4. Adding graphic message with cheques.

9.1 Credible source	Present verbal or visual communication from a credible source in favour of or against the behaviour	1. Dissemination of information via<br>a) Safety advisors<br>b) Medical professionals<br>c) Farm representatives<br>d) Reputed media channels<br>e)Equipment dealers
10.8 Incentive(outcome)	Inform that a reward will be delivered if and only if there has been effort and/or progress in achieving the behavioural outcome	1. Financial incentives<br>a) for retrofitted tractors<br>b) completing intervention related tasks
10.11 Future Punishment	Inform that future punishment or removal of reward will be a consequence of performance of an unwanted behaviour	1. Non-compliance lead to the removal of access or privilege in future to<br>a) operate a vehicle<br>b) Financial support
13.1. Identification of self as a role model	Inform that one's own behaviour may be an example to others.	1. Encourage participants to set a positive example for family members / workers by adopting specific safety behaviours.
13.3 Incompatible beliefs	Draw attention to discrepancies between current or past behaviour and self-image, in order to create discomfort	1. Create situations to draw attention to safety beliefs and risky habits to induce cognitive dissonance

16.3 Vicarious consequences	Prompt observation of the consequences for others when they perform the behaviour	1. Demonstrates the consequence of accidents with others<br>2. Narrate the accidents happened to others and its impacts<br>3. Accident survivors sharing their experience
